# A Reassessment of Tryptophan Requirements for Aged Brown Laying Hens Using Amino Acid Biomass

**DOI:** 10.3390/ani16050723

**Published:** 2026-02-25

**Authors:** Lívia Rastoldo R. Oliveira, Stephane C. O. Estevão, Michele Bernardino de Lima, Rita Brito Vieira, Larissa Oliveira dos Santos, Tiago Araujo Rodrigues, Bernardo Rocha F. Nogueira, Edney Pereira da Silva

**Affiliations:** 1 Department of Animal Sciences, School of Agricultural and Veterinary Sciences, São Paulo State University (UNESP), Jaboticabal 14884-900, SP, Brazil; livia.r.oliveira@unesp.br (L.R.R.O.); s.estevao@unesp.br (S.C.O.E.); rita.brito@unesp.br (R.B.V.);; 2 Department of Animal Production and Health, School of Veterinary Medicine, São Paulo State University (UNESP), Araçatuba 16050-680, SP, Brazil; michele.bernardino@unesp.br; 3 CJ Bio Brazil, São Paulo 04571-010, SP, Brazil; bernardo.nogueira@cj.net

**Keywords:** efficiency utilization, egg production, supplementation technique

## Abstract

A reassessment of the Trp requirements for brown laying hens using the Trp biomass source allows an update of the nutritional recommendation, in addition to assessing whether biomass has an adjuvant effect in the diet. This research was carried out with the aim of evaluating the efficiency utilization of Trp and determining the ideal Trp intake for aged brown laying hens using the L-Trp biomass 60%. A completely random design was used, with eight treatments and eleven replicates, totaling 264 hens. The diets were formulated by supplementation technique using one basal diet with 0.112% of digestible Trp, resulting in amino acid levels that ranged from 0.112 to 0.225% of digestible Trp. The variables analyzed were performance and egg quality. When the effect of Trp levels (*p* ≤ 0.05) was detected, the model’s linear–plateau (LP), quadratic–plateau (QP), and the first intercept of the QP in the plateau of LP were adjusted to determine the ideal Trp intake. The results obtained show an effect of the treatments (*p* < 0.05) only on the performance variables. The first intercept results obtained were 186.2, 174.7, 182.7, and 182.5 mg/hen per day for egg production, egg mass, feed conversion ratio, and feed efficiency, respectively. Dietary supplementation with L-Trp biomass 60% effectively meets the nutritional requirements of aged brown laying hens, supporting efficient nutrient utilization without impairing productive performance or egg quality.

## Simple Summary

Animal cells cannot synthesize tryptophan (Trp), and metabolic intermediates such as indole-3-pyruvic acid provide only minimal and metabolically insufficient conversion to Trp, reinforcing its classification as an essential amino acid in vertebrates. This study assesses the tryptophan requirements of aged layers using an L-Trp biomass of 60%. A total of 264 hens received eight Trp levels, and performance and egg quality were measured. When significant effects were found, linear–plateau and quadratic–plateau models estimated optimal intake. Trp influenced only performance variables, with ideal intake values ranging from 174.7 to 187 mg/hen per day. Based on Trp intake and its deposition in eggs, the optimal requirement was determined to be 187 mg/hen per day for maximum efficiency.

## 1. Introduction

The molecular structure of tryptophan (Trp) was initially characterized through its synthesis from indole-3-aldehyde [1]. In microorganisms and plants, Trp is biosynthesized via the shikimate pathway, a metabolic route shared by aromatic amino acids, in which chorismate serves as the final common precursor. Vertebrates are unable to synthesize aromatic amino acids due to the absence of this pathway, which mediates the conversion of phosphoenolpyruvate and erythrose-4-phosphate into chorismate [2]. Although these precursors are generated through gluconeogenesis [3] and the pentose phosphate pathway [4], vertebrates lack the genes encoding DAHP synthase and chorismate synthase, enzymes essential for chorismate formation [5]. Animal cells cannot synthesize tryptophan, even in the presence of intermediates such as 3-indole aldehyde, L-β-3-indolelactic acid, or kynurenine [6,7]. Among these, only indole-3-pyruvic acid (IPA) can be converted to Trp via transamination, although this route is quantitatively constrained [8,9,10].

While tryptophan was recognized as essential in the 1930s [7,8,9], dietary supplementation for poultry began only in the 1950s [11]. Since then, biotechnological processes using *Escherichia* and *Corynebacterium* strains have enabled large-scale L-tryptophan production for animal feed [12,13,14]. The characterization of novel L-tryptophan sources should encompass assessments of digestibility [15], bioavailability [16], and utilization efficiency, particularly for biomass-derived forms, whose matrix may influence absorption and metabolism. Moreover, methodological differences related to classical studies warrant reassessment of Trp requirements in brown laying hens. In this framework, biomass-derived Trp enables updating nutritional recommendations and evaluating potential adjuvant effects of the associated matrix on supplemental L-tryptophan.

Three studies in brown laying hens [17,18,19] recommended Trp intakes of 171, 235, and 217 mg/day, spanning a 64 mg/day range (37% variation), with only [17] clearly reporting egg mass responses to dietary Trp restriction. Utilization efficiency data from [17,18,19] showed a strong negative linear relationship between Trp intake (I) and efficiency (k) (k = 104 − 0.246 × I; R^2^ = 0.899), indicating that higher Trp intake does not proportionally increase Trp deposition. Consistently, ref. [19] reported an efficiency of ~23% (k = 93.6 − 0.2316 × I; R^2^ = 0.869), implying that ~75% of ingested Trp was redirected to non-egg metabolic pathways. Accordingly, alternative indicators such as feather condition and stress metrics were used [18,19]; however, these reflect maintenance processes, and Trp allocation to maintenance could not be quantified due to missing body weight data [17,18,19]. Despite Trp’s role in basal metabolism, ref. [20] estimated egg production utilization efficiency at 55.4% in heavy broiler breeder hens, exceeding values observed in commercial brown layers.

Therefore, the hypothesis that a higher Trp utilization efficiency for egg-protein deposition occurs in layers is supported by laying sequence length: breeders have shorter laying sequences and longer pauses between sequences compared to commercial layers [21]. Considering the above, this study was undertaken to evaluate Trp utilization efficiency and to determine the ideal Trp intake for aged brown laying hens using a 60% biomass L-tryptophan source.

## 2. Materials and Methods

### 2.1. Ethical and Study Approvals

A study was conducted using brown laying hens at the Poultry Science Laboratory of São Paulo State University (UNESP, Jaboticabal, São Paulo, Brazil). All experimental procedures were approved by the Animal Ethics and Welfare Committee of UNESP under protocol number 3340/22, approval on 15 June 2022.

### 2.2. Housing, Experimental Design, and Management

A total of 264 Brown Hy-Line hens were housed in the experimental facilities, an open shed in a two-tier cage system, equipped with linear feeders, nipple drinkers, and six fans. At 68 weeks of age, hens were selected based on body weight (1.760 ± 0.09 kg) and egg production (91 ± 2%) and were distributed into 88 experimental units. The egg production from each experimental unit was monitored for two weeks to ensure maximum uniformity. Before the start of the experiment, Levene’s and F tests were applied to verify the homogeneity of variances in egg production and body weight between treatments. The experimental design was completely randomized, with eight treatments and 11 replicates of three hens each. The lighting program was 16 h of light, using an intensity of 30 lux. The experiment lasted 16 weeks. Temperature and humidity were recorded daily, and their average values for maximum and minimum were approximately 30.67 °C, 16.09 °C, and 70.96%, and 36.21%, respectively. The hens were fed 110 g of feed/day, and water was provided *ad libitum*.

### 2.3. L-Tryptophan Biomass 60%, Diets and Treatments

The source of L-tryptophan biomass 60% used in this study contains an amount of biomass contained in the product, which varies from 25% for threonine and 30% for valine to 40% for tryptophan. L-tryptophan biomass is produced through fermentation, and granules are formed after steam heating. The biomass, which corresponds to approximately 40%, contains protein, essential amino acids, organic acids, and carbohydrates [16].

### 2.4. Diets and Treatments

A basal diet (BD) was formulated to meet the nutritional requirements for metabolizable energy, minerals, vitamins, protein, and essential amino acids, except for Trp, which was defined as the first limiting amino acid in the dietary profile protein. The contents of metabolizable energy, digestible amino acids, available phosphorus, and total calcium of the ingredients—corn, soybean meal, and meat and bone meal—were determined using near-infrared spectroscopy and used to adjust the experimental diet formulations.

The Trp level of 0.112% g/kg in the diet was obtained without a source of crystalline L-tryptophan (Table 1). The BD was used as treatment 1, with the first level being the Trp—D1 (Table 1). The treatments consisted of eight Trp levels obtained by the supplementation technique (Table 2). The other treatments were obtained with the supplementation of the L-tryptophan biomass 60% in the BD (Table 2). To balance crude protein and energy in the diets, kaolin, L-glutamic acid 98%, and starch were used (Table 2). The levels were D1: 0.112%, D2: 0.127%, D3: 0.142%, D4: 0.157%, D5: 0.172%, D6: 0.187%, D7: 0.202%, and D8: 0.225% of digestible Trp (Table 3). Recommendations for nutritional requirements were obtained from [22], as follows: 2.850 kcal/kg of metabolizable energy corrected; 15.76% of crude protein; 0.875% of digestible lysine; 0.858% of digestible methionine+ cysteine; 4.5% of total calcium; 0.332% of available phosphorus and other nutrients as presented in Table 1. The main feed ingredients were analyzed for amino acid content by near infrared spectroscopy using the AMINONIR service of Evonik Nutrition & Care GmbH (Essen, Germany). The analyzed amino acid values were subsequently used for diet formulation.

### 2.5. Experimental Procedures and Performance Variables

The body weight was measured at the beginning and end of the assay. Weekly, the leftovers were weighed to quantify feed intake. Egg production and mortality were recorded daily. Egg weight was measured on two consecutive days per week. The variables analyzed were daily feed intake (FI, g/hen d), body weight (BW, g), daily egg production (EP, %), egg weight (EW, g), daily egg mass (EM, g/henꞏday), feed conversion ratio by egg mass, corrected for mortality (FCR, g/g), change in body weight (cBW, g/hen), Trp intake (I, mg/hen day), tryptophan deposition in egg (D, mg/henꞏper day), and Trp maintenance (m, mg/henꞏper day).

The total amount of protein in an egg, the percentage of Trp of this protein, and the total Trp deposited in an egg were calculated using the concentration of protein (6.25 × 451 mg of nitrogen per g of dry matter) and Trp deposited in eggs of hens (108 mg/g nitrogen in an egg) obtained from [23]. The dry matter value in the egg was obtained from [24]. The average value of 10.4 mg of Trp for maintenance per metabolic body weight (kg^0.67^) was obtained by [25,26].

### 2.6. Egg Assessment and Quality Variables

At the end of the trial, two eggs per experimental unit, totaling 22 eggs per treatment, were randomly selected for egg quality analysis. The eggs were evaluated for egg weight, albumen height, yolk weight, and eggshell thickness. The variables analyzed were yolk weight (YW, g), eggshell weight (ESW, g), albumen weight (AW, g), eggshell thickness (EST, μm), percentage yolk (PY, %), percentage albumen (PA, %), and percentage shell (PS, %). The eggs were weighed and broken; the yolk was separated from the albumen and shell. The shells were washed and air-dried for 48 h. They were then weighed, and the thickness was measured. The weight of the albumen was considered as the difference between egg weight and the sum of the weight of the yolk + shell. The egg weight, yolk weight, and eggshell weight were determined using an analytical balance (Model: 2202H BEL, BEL Engineering, Monza, Italy). The thickness was measured with a caliper (0–150 mm; model 100-174BL, Digimess, São Paulo, Brazil).

### 2.7. Cumulative Effect of Dietary Trp on Body Composition

At the end of the trial, a hen with a BW matching the average of the experimental unit was selected for body composition analysis using dual-energy X-ray absorptiometry (DXA) technology, totaling 88 hens. The hens were submitted to a solid 8 h of fasting for emptying of the digestive tract, then they were weighed for body composition analysis, total mass, area, bone mineral content, bone mineral density, fat mass, and lean using DXA technology. Based on the equations by [27], the body composition in protein, lipid, water, and ash was obtained. The variables analyzed were body water (g/hen), body protein (g/hen), body lipid (g/hen), and body ash (g/hen) of the hens.

### 2.8. Models to Response Interpretation

The models were adjusted using Trp intake (mg/henꞏper day). The value in the abscissa corresponding to the first intercept of the quadratic curve of the linear–plateau model was also calculated to determine the ideal Trp intake. The adjusted models were:

LP: γ = φ + α[λ − δ] (1)

where (λ − δ) is 0 for δ values > λ, γ is the response, δ is the regressor variable, φ is the corresponding response to λ on the orderly axis, λ is the point that indicates a change in the trajectory of γ corresponding to the axis of the abscissas, and α is the slope.

QP: γ = ξ + β[τ − δ]^2^ (2)

where (τ − δ) is 0 for δ values > τ, γ is the response, δ is the regressor variable, ξ is the corresponding response to τ on the orderly axis, τ is the point that indicates a change in the trajectory of γ corresponding to the axis of the abscissas, and β is the slope. The value in the abscissa corresponding to the first intercept of the QP curve on the plateau of the LP model (QP_1_–LP) was calculated as follows:

QP_1_–LP: δ = −(−βλ + sqrt (βφ − βλ))/β (3)

For this broken line two-slope or linear–linear (LL), there was only a relation between Trp deposition in the egg and Trp intake, according to the following equation:

LL: γ = φ + α[λ − δ] + β[δ − λ] (4)

where φ represents the coordinate on the ordinate axis and λ on the abscissa axis of a breakpoint on a curve. α is the slope coefficient of a line when δ < λ, and in the two-slope model, β represents the slope of a line when δ > λ. So, by definition, (λ − δ) is zero when δ > λ and (δ − λ) is zero when δ < λ.

The total or gross efficiency of Trp utilization (k_t_) was obtained from the ratio of φ to λ for the LP model and ξ to τ for the QP model, based on parameters fitted to EM, using Equations (1) and (2). The k_t_ for QP_1_–LP was obtained from the ratio of δ, the QP_1_–LP, and φ, the LP model. The efficiency of Trp utilization in egg deposition (k_e_) was obtained based on Equation (4), using the ratio of φ to λ for the LL model.

### 2.9. Statistical Analysis

The data were tabulated and analyzed for the assumptions of homoscedasticity of variance (Brown–Forsythe) and normality of errors (Cramér–von Mises) using a mixed model. The experimental unit was considered as the random effect, and Trp levels as the fixed effect. The variables were subjected to orthogonal contrast analysis for linear and quadratic effects of Trp levels. When an effect was detected, considering a significance at 0.05 (*p* ≤ 0.05), regression analysis was applied using the broken-line (BL) models: linear–plateau (LP), quadratic–plateau (QP), and linear–linear (LL), according to [28]. All data were analyzed using the MIXED SAS (PROC MIXED) procedure within the SAS 9.4 software (Statistical Analysis for Windows, SAS Institute Inc., Cary, NC, USA).

## 3. Results

### 3.1. Performance, Egg Quality, and Body Composition

The average values of FI, EP, EW, EM, FCR, and FF of hens fed different levels of Trp in the diet from 70 to 86 weeks of age are presented in Table 4. According to the probability values for F-statistics of ANOVA, linear and quadratic effects (Table 4), Trp levels did not affect FI and EW (*p* > 0.05). EP was affected by the Trp level in the diet, and the other variables obtained through the relationship with EP were also affected (Table 4), such as EM, FCR, and FF (*p* < 0.01).

The maximum and minimum response values for EP were 94.6% and 81.4%, obtained with 0.187% and 0.112% of Trp in the diet, respectively. Resulting in an amplitude of 13.2% units in EP, which is equivalent to approximately 14% reduction in EP in relation to the maximum EP value, obtained with 0.187% of Trp in the diet (Table 4). This percentage of reduction or modification in the response due to Trp deficiency in the diet remained in the other variables obtained from EP, such as EM, FCR, and FE, with 15.0%, 14.5%, and 14.5% reduction in relation to the maximum value, respectively. It is important to highlight the experimental variability; the coefficient of variation (CV) for FI and EW was low, while the CV for EP and other variables obtained using EP was three times the CV for FI and twice that for EW (Table 4).

The average values of YW, ESW, AW, EST, PY, PA, and PS of hens fed different levels of Trp in the diet from 70 to 86 weeks of age are presented in Table 5. The results obtained for the egg quality variables support the statement that Trp levels did not affect (*p* > 0.05). any of the variables analyzed after 16 weeks of experimentation. The values of CV of the variables were low (Table 5).

Average responses of BW, cBW, and major body chemical components—water, protein, lipid, and ash—of hens fed different levels of dietary Trp from 70 to 86 weeks of age are presented in Table 6. There was no significant effect (*p* > 0.05) of Trp levels on BW and cBW (Table 6). For the chemical composition of water, protein, and ash in the body, a significant effect was detected for the F test (*p* < 0.01) of Trp levels, but it was not possible to model the effect with linear and quadratic adjustment; both were non-significant (*p* > 0.05). The body lipid (γ) content was significant for the F test, linear and quadratic effect (Table 6) of Trp levels (δ) in the diet. Considering the highest degree polynomial, that is, the quadratic effect (γ = −2930.2δ^2^ + 899.9δ + 108.83, R^2^ = 0.3381), the body lipid presented two minimum points, coinciding with the lowest (0.127% of Trp) and highest (0.225% of Trp) levels of Trp in the diet, and a maximum point, close to the level of 0.172% of Trp in the diet.

### 3.2. Models for Response Interpretation to Estimate Dietary Tryptophan Requirement

According to Table 7, both the LP and QP models estimate a maximum response of 94.4% for EP. The LP model identifies a breakpoint at 180.9 mg/hen, while the QP model estimates it at 207.3 mg/hen. The R^2^ values for both the LP and QP models were similar, at 78% (Table 7). A distinction between the models was observed only in the model selection statistic, AIC, where the LP model had the lower value (343.3) compared to the QP model (347.6). The QP_1_–LP occurs at 186.2 mg/hen, corresponding to 89% of the QP model’s estimated breakpoint (Table 7).

The estimated maximum response was 56.1, 1.921, and 520.2 for EM, FCR, and FE using LP and 56.2, 1.923, and 520.2 for EM, FCR, and FE using QP, respectively. The coefficient of determination (R^2^) indicated similar goodness of fit between the LP and QP models for each response variable (Table 7). Differences in R^2^ values between models, LP and QP, were minimal, with variations of 0.7, 0.2, and 0.2 for EM, FCR, and FE, respectively. However, model selection based on the Akaike information criterion (AIC) favored the LP model, which consistently exhibited lower AIC values across all variables. The relationship between the Trp recommendations in the diet estimated by LP was close to 89% of the value found for QP. Meanwhile, the QP_1_–LP was 90%, therefore very close to the value estimated by LP (Table 7). Based on the dose–response analysis, the Trp level of 186.2 mg/hen, as estimated by QP_1_–LP to EP, can be recommended as it meets the requirements for the remaining response variables (Table 7).

### 3.3. Efficiency of Tryptophan Utilization for Egg Deposition

Based on the adjusted parameters for EM estimated using the LP, QP, and QP_1_–LP, as presented in Table 7, the values of kₜ were calculated. The estimated kₜ values were 0.63 or 63% for the LP model, 0.56 or 56% for the QP model, and 0.61 or 61% for the QP_1_–LP model. The average values of Trp intake, Trp deposited in eggs, and Trp used for maintenance of hens fed different levels of Trp in the diet from 70 to 86 weeks of age are presented in Table 8. According to the probability values for F-statistics of ANOVA, linear and quadratic effects (Table 8), Trp levels did not affect the Trp used to maintain the BW of the hens (*p* > 0.05). The average amount of tryptophan allocated for maintenance was 15.6 mg/hen per day, regardless of dietary Trp concentration.

The amount of Trp required per gram of egg mass, expressed in mg of Trp per g of egg, was calculated, and the values were 3.0 and 2.71 for the LP model, 3.36 and 3.07 for the QP model, and 3.11 and 2.82 for QP_1_–LP, respectively, to total and net utilization of the amount of Trp required per gram of egg mass.

The variables Trp intake and Trp deposited in the egg were affected by Trp levels, showing linear and quadratic effects (*p* < 0.05). The maximum and minimum response values for Trp deposited in the egg were 107.2 mg/hen and 91.0 mg/hen, obtained with 0.187% and 0.112% of Trp in the diet, respectively. Resulting in an amplitude of 16.2 mg/hen when the Trp was deposited in the egg, which is equivalent to approximately 15.1% reduction in Trp deposited in the egg in relation to the maximum value obtained with 0.187% of Trp in the diet (Table 8).

**Figure 1:**
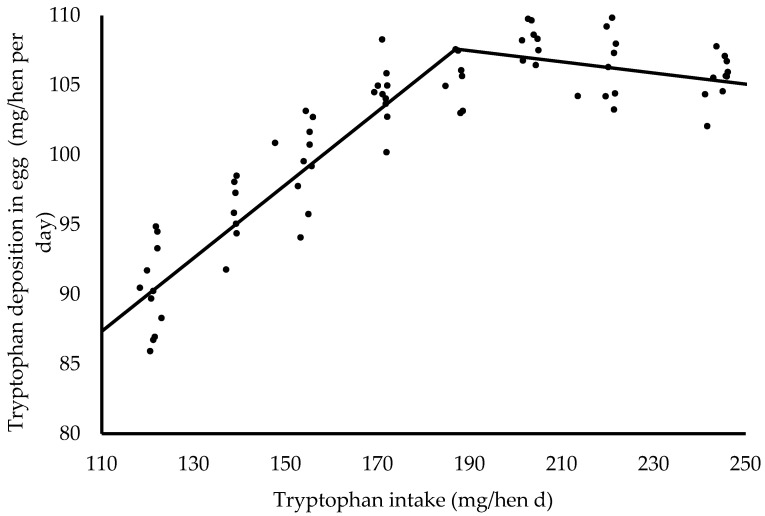
Relationship between tryptophan intake and tryptophan deposition in eggs of hens from 70 to 86 weeks of age. Adjusted equation: γ = 107.6 − 0.263[187 − δ] − 0.04[δ − 187], R^2^ = 95%.

The relationship between Trp intake and Trp deposited in the eggs of hens was adjusted by the equation γ = 107.6 − 0.263[187 − δ] −0.04[δ − 187], R^2^ = 95%, Figure 1. Based on model LL, the Trp intake of 187 mg/hen per day, as estimated to maximize Trp deposited in egg, can be recommended as it meets the requirements for the remaining response variables (Figure 1), including EP. The k_t_ was obtained using the ratio of φ = 107.6 mg/hen to λ = 187 mg/hen, resulting in 0.575 or 57.5%. Taking the reciprocal of k_t_, 1/k_t_, the value obtained was 1.74 mg Trp/g egg mass. The values of k_e_ and 1/k_e_ were obtained based on considering only partitioning to Trp deposited in the egg; therefore, the Trp intake used for maintenance was subtracted (Trp intake for deposition is 171.4 mg/hen). The value of k_e_ was 0.627 or 62.7% and 1/k_e_ was 1.59 mg of Trp per g of Trp deposited.

## 4. Discussion

Due to the limited number of studies involving brown egg-laying hens, this section includes relevant research conducted with both white and brown Leghorn strains. Previous studies reported no effect of dietary tryptophan (Trp) on feed intake or egg weight [29,30]. Dong et al. [31] reported that egg weight could be related to the inadequate amount of one or more other amino acids, but since the amino acids in the diet were within the recommended levels for laying hens, including Trp at different levels, such differences may not have been found [32,33].

Daily egg production showed a linear or quadratic–plateau response, consistent with previous reports [33,34,35]. Although the exact mechanisms remain unclear, tryptophan may enhance egg production as a precursor of melatonin, which supports ovarian cell regeneration, follicular development, and ovulation [36].

Similarly, egg mass, feed conversion ratio, and feed efficiency showed both linear and quadratic responses to increasing dietary tryptophan (Trp) levels (Table 4). Optimal egg mass was obtained from 175 mg/hen per day (Table 7). Previous research has also indicated that adequate Trp intake supports optimal egg mass in laying hens during the late production phase [30]. Similar observations have been reported regarding egg mass and feed conversion ratio in previous studies [32,33].

Adequate tryptophan (Trp) intake is important for follicular development and may influence yolk proportion [37]; however, the lack of differences in yolk weight indicates that even the lowest dietary Trp levels were sufficient. Consistent with these findings, other studies reported no significant effects of Trp on yolk weight, eggshell thickness, or albumen weight [33,38,39,40]. Although melatonin, a Trp-derived metabolite, may enhance calcium absorption and potentially improve eggshell quality [41], the mechanisms by which dietary Trp modulates calcium metabolism and eggshell formation remain unclear.

The egg is composed primarily of yolk, albumen, and eggshell with membrane, which represent approximately 30%, 60%, and 10% of total egg weight, respectively [42]. In the present study, dietary Trp levels did not influence the relative proportions of yolk, albumen, or eggshell (Table 5).

A positive linear effect was observed for Trp intake as dietary tryptophan levels increased (Table 8). The partitioning of Trp toward maintenance requirements was also influenced by dietary Trp levels (Table 8). This outcome was expected, as maintenance requirements are closely associated with the hens’ metabolic weight, which is calculated as body weight raised to the 0.67 power. Notably, body weight itself was not affected by dietary Trp levels (Table 6). The Trp deposition exhibited both linear and quadratic responses (Table 8), with a maximum deposition of 107.6 mg/hen per day observed at a dietary intake of 187 mg/hen per day (Figure 1). This response pattern reflects an increase in Trp deposition as dietary levels approach the hens’ physiological requirement, followed by a decline when intake exceeds this threshold [43,44].

This research was conducted to evaluate the efficiency of Trp utilization and determine the ideal Trp intake for aged brown layers using an L-tryptophan biomass 60% source. Based on the results obtained, it was possible to extract values of Trp utilization efficiency for aged brown layers and derive nutritional recommendations for Trp in the diets of these hens.

The values of k_t_ and k_e_ were determined to be 61% and 63%, respectively. These indices refer to the efficiency with which the aged layers utilized Trp, denoting the fraction used for maintenance and egg production (k_t_), whereas represents the fraction of ingested Trp destined for protein deposition in the egg. These results support the hypothesis formulated in this research that commercial layers are more efficient than broiler breeders’ hens [20,45,46].

Therefore, the higher value of utilization efficiency observed in commercial layers is supported by the length of the laying sequence of these birds, since heavy-type breeders have shorter laying sequences [21] and longer pauses [21] between sequences compared with commercial layers. This genetic condition favors greater utilization for egg production, especially at the 94.6% egg production level observed in this study.

The efficiency value (k_e_) for tryptophan was lower than that reported for other amino acids, such as lysine, methionine  +  cysteine, and threonine [47,48]. This may be explained by the multifunctional role of Trp in physiological processes beyond egg protein deposition, reducing its recovery in the egg despite its importance for bird health [49]. Additionally, methodological factors, particularly the relatively mild dietary Trp deficiency used in some studies, may have limited the detection of effects on egg protein synthesis, as laying hens can mobilize body reserves to maintain production, thereby influencing ke estimates [18,19,20,45,46,47].

In this research, the 15% deficiency was sufficient to produce an effect on egg production after 16 weeks, among directly measured variables such as feed intake and egg weight. Therefore, aged brown or semi-heavy layers have body reserves, as indicated by cBW responses (Table 6). Body reserve capacity was analyzed considering its influence on the adaptation period of the requirement study [50]. Those authors used laying quails, an animal model with low body reserve capacity, and a diet with a 20% deficiency of the first limiting amino acid; at the end of the study, they recommended 28 days as sufficient to impose deficiency and derive results. Finally, the degree of limiting amino acid deficiency and the birds’ physiological state should be considered to establish dietary levels and the experimental period to obtain clear responses for feed intake, egg weight, and egg quality, which were not observed in this study.

In addition, the study by [17] imposed a 32% deficiency in commercial layers at the beginning of the laying peak (31–37 weeks) with a 42-day experimental period and obtained clear responses for feed intake, production, and egg weight. The results obtained by [17] showed that the birds mobilized body reserves to the point of recording negative cBW values, demonstrating that the physiological status of young birds is greatly influenced by the effects of dietary deficiency.

From the results of [17], a k_e_ value of 58.6% was derived. Based on this value, it is apparent that the birds in the present study were more efficient in the use of dietary Trp. The higher efficiency observed in this study is related to the potential for egg production: the maximum egg production achieved here was 94.6%, whereas in the study of [17] it did not exceed 85%. Despite the difference in age of the birds between 70 and 86 weeks (present study) versus 31–37 weeks [17], in this study the birds exhibited longer laying sequences and shorter pauses between laying sequences. This underscores the importance of constantly reevaluating nutritional requirements, given the improvements in the genetic progress of modern birds.

Traditionally, amino acid requirements for laying hens have been estimated using linear regression of empirical data or by developing partitioning models based on assumed linear relationships between nutrient intake and output [25,51,52]. The estimation of Trp requirements per unit of egg output, expressed as mg Trp per g egg mass, was based on this linear assumption. Different response functions (linear and non-linear) were exhaustively evaluated [44]. It was observed that the point marking the end of the linear response phase represented a region where all models converged toward the mean estimated requirement [44]. Beyond this point, divergence among functions became more evident, depending on the specific parameterization of the asymptotic response [44].

Comparison based on 1/k_e_ values eliminates the confounding effect of maintenance requirements on the utilization of Trp for egg mass production. The estimates derived from the combination of the QP_1_–LP and LL models were 2.82 and 1.54 mg/g, respectively. Although obtained through different modeling approaches, these values exhibit comparable predictive capacity, as both are grounded in the same efficiency of utilization. The key distinction lies in the input variable: QP_1_–LP uses grams of egg mass, a direct measurable output, whereas LL employs the amount of tryptophan deposited (mg). Previous studies have typically used egg mass as the input variable [25,51,53].

Pioneering studies reported a requirement of 2.25 mg of Trp per gram of egg mass [25,53]. Unlike the present study, those authors estimated Trp requirements for both egg mass production (2.25 mg/g) and body weight maintenance (10.25 mg/kg) simultaneously, which introduced a correlation between input variables—egg mass and body weight. This correlation influenced the intersection point of the response curves and, consequently, the partitioning estimate for maintenance. In the present study, the maintenance requirement was fixed based on literature values (10.4 mg/kg^0.67^) and subtracted from total intake to isolate the Trp requirement for egg mass production: [(174 mg − 16.4 mg)/56.1 g].

To reduce the correlation between input variables and improve the reliability of estimates, some studies have employed datasets compiled from multiple experiments. Using this approach, ref. [51] reported a requirement of 2.62 mg Trp/g of egg mass. In the current study, the net Trp deposition per gram of egg mass was estimated at 1.89 mg/g. Therefore, kₑ can be calculated as the ratio of deposition to intake. Based on this approach, k_e_ values extracted from [25,51] were 84% and 71%, respectively.

The differences in k_e_ between these studies and the current work are likely attributable to differences in hen strain. The present study uses brown-egg layers, which are less efficient than the light hybrids used in previous studies. The 1/k_e_ values determined here can be applied in factorial models to support nutritional recommendations beyond the specific experimental conditions of this trial.

A secondary objective of this research was to determine the ideal intake of Trp for aged brown laying hens using the L-tryptophan biomass 60% source. The methodology applied in this research, considering the formulation of experimental levels and the experimental period, was sufficient to modify the hens’ responses, allowing productive responses to be obtained (Table 4), which supported the derivation of nutritional recommendations for Trp in the diet for these birds. Based on the results obtained, the ideal Trp intake is 187 mg/hen per day. This intake was defined following the highest value criterion to meet the requirements of other variables, especially egg production, which was estimated at 186.2 mg/hen per day, close to the value obtained from the relationship between deposition and Trp intake (Figure 1).

The responses to increasing dietary Trp levels were fitted to linear–plateau, quadratic–plateau, and linear–linear models to interpret the data and derive nutritional recommendations. The parameters λ and τ indicate the change point in Trp intake and were considered as the ideal intake, with τ values averaging 13% higher than λ (Table 7). Therefore, an intermediate Trp intake between λ and τ was calculated using the φ value from the linear–plateau model in the quadratic equation. The resulting estimates (186.2, 174.7, 182.7, and 182.5 mg/hen per day for egg production, egg mass, feed conversion, and feed efficiency, respectively; Table 7) were close to the 187 mg/hen per day estimated from Trp deposition, supporting a robust recommendation based on multiple statistical approaches [54].

The recommendation proposed by [18] was approximately 33% higher than the value defined as the recommendation in this research. The difference between the recommendations is related to the utilization efficiency, which in this study was 63%, while the efficiency value of [18] was 46%; therefore, to produce 53 g/day, the laying hens from [18] required an intake of 249 mg Trp/hen per day.

A study with Hy-Line Brown hens (70–74 weeks) recommended a Trp intake of 209–225 mg/hen per day to produce 47.5–48.7 g/day of egg mass, based on egg production (72–75%) and egg weight (64–65 g) over four weeks [19]. From this study, Trp utilization efficiency ranged from 44% to 46%, assuming 15.5 mg for maintenance. The higher efficiency observed in the present research is attributed to greater genetic potential for egg production (94.6% vs. ≤75% in [19]). Although hen age was similar, the longer experimental period (16 weeks) and the imposed dietary Trp deficiency allowed a more accurate estimation of Trp effects and requirements. Consequently, the Trp recommendation obtained here was higher than the 171 mg/hen per day reported by [17], reinforcing the need to update Trp requirements for brown laying hens.

The recommendation established in the present study (187 mg/hen per day) is based on an average bird with a body weight of 1.830 kg and a daily egg mass production of 56.7 g/hen. This recommendation was lower than that presented by [22] of 201 mg/hen per day and close to the updated recommendation in 2024 of 187 mg/hen per day, considering the average described in this research. The systematic review conducted by [55] resulted in an average of 167 mg/hen per day, obtained from eight publications that used Leghorn, white, or brown hens.

According to [56], dietary Trp has a greater effect in the late laying period, attenuating age-related declines in egg production. This may be linked to its interaction with estradiol, which increases with age [57] and is negatively associated with egg production through its relationship with plasma progesterone [58]. Elevated estrogen levels may contribute, though not exclusively, to reduced egg production in aged layers [59]. Improved performance may result from reduced estrogenic activity or mitigation of its adverse effects on Trp metabolism via increased plasma Trp. Estrogens can also modulate enzymes involved in Trp metabolism, such as tryptophan oxygenase and kynureninase [60,61,62].

This study evaluated a new tryptophan source, L-tryptophan biomass 60%, complementing previous digestibility [5] and bioavailability studies [6]. Requirement trials, which use higher supplementation levels and longer experimental periods, support the assessment of new amino acid sources. No adverse effects were observed with this product. Reassessing Trp requirements for brown layers enabled updated nutritional recommendations and evaluation of any additional effects of the biomass component, which is produced by fermentation and steam granulation. The product contains about 40% biomass, including protein, essential amino acids, organic acids, and carbohydrates [63], justifying further evaluation in poultry diets.

The results in this research support the analysis of some hypotheses related to the contribution of biomass to amino acid utilization for egg production. The first hypothesis that can be evaluated regarding L-tryptophan biomass 60% concerns the presence of some compound that may have an action or act as an antinutritional factor. Analyzing the laying hens responses, there was no effect from the treatments, especially for feed intake and egg weight, which were statistically equal, ruling out any negative effect from increasing the inclusion of the L-tryptophan biomass 60% source on the laying hens responses.

The second hypothesis concerns the presence of some factor in the biomass that could positively contribute to improving the laying hens’ responses. A precise analysis rules out the possibility of mobilizing body reserves to maintain egg production since there was an improvement in production with positive responses for cBW without association with Trp levels in the diet.

The third hypothesis addresses Trp digestibility in laying hens, assuming 100% digestibility of L-tryptophan biomass 60%. Although Trp excretion was not measured, the absence of negative effects on performance (Table 4) and egg quality (Table 5) at the highest Trp levels supports this assumption. The granular form of L-tryptophan biomass 60% may slow gastrointestinal transit and enhance Trp absorption and utilization. Nonis and Gous [64] observed that for each additional gram of free amino acids per kg of feed, egg production and egg mass decreased by 32.5 g per day, respectively. This means that free ‘synthetic’ amino acids are not as well utilized as protein-bound amino acids when birds are fed a restricted amount of feed. Although not measured, future studies should evaluate passage rate to confirm the effects of the granular form on gastrointestinal dynamics.

## 5. Conclusions

This study demonstrates that dietary supplementation with 60% L-tryptophan is an effective and safe strategy to meet the nutritional requirements of aged brown laying hens. Adequate L-tryptophan biomass (60% supply) supports efficient nutrient utilization without compromising productive performance or egg quality. The results indicate that hens at the end of the laying cycle are able to maintain high productive efficiency when their amino acid requirements are properly balanced. These findings contribute to refining nutritional recommendations for tryptophan and reinforce its importance in sustaining performance during the late laying phase.

## Figures and Tables

**Table 1:** Feed composition and nutrient composition calculated for the basal diet (g/kg).

Ingredients	Basal Diet
Grain Corn	715.947
Soybean Meal 46%	121.925
Meat and bone meal 42%	47.413
Fine Limestone	14.578
Coarse Limestone	72.146
NaCl	3.120
L-Met 100%	2.823
L-Lysine 78%	2.713
L-Threonine 98%	1.141
L-Valine 96%	0.922
L-Isoleucine 98%	1.460
L-Arginine 98%	0.458
Choline Chloride 60%	0.710
Lignocellulose	5.064
Premix ^1^	1.000
L-Glutamic acid 98%	2.518
Starch	1.000
Kaolin	5.063
Total	1000
**Nutrient composition calculated**	
Dry matter, %	88.836
Crude fiber, %	2.198
Crude protein, %	14.560
Metabolizable energy correct, kcal/kg	2825
Digestible Lysine, %	0.746
Digestible Methionine, %	0.487
Digestible Methionine + Cysteine, %	0.683
Digestible Threonine, %	0.525
Digestible Tryptophan, %	0.112
Digestible Arginine, %	0.777
Digestible Glycine + Serine, %	1.255
Digestible Valine, %	0.651
Digestible Isoleucine, %	0.599
Digestible Leucine, %	1.151
Digestible Histidine, %	0.322
Digestible Phenylalanine, %	0.577
Digestible Phenylalanine + Tyrosine, %	1.018
Potassium, %	0.478
Sodium, %	0.160
Clorine, %	0.354
Total calcium, %	4.000
Available Phosphorus, %	0.345
Total Choline, mg/kg	1186
Linoleic Acid, %	1.473

^1^ Content per kg of diet: folic acid—1.232 mg; pantothenic acid—26.40 mg; biotin—0.0352 mg; niacin—61.60 mg; vitamin A—4.224 mg; vitamin B1—3.872 mg; vitamin B12—0.02112 mg; vitamin B2—10.032 mg; vitamin B6—5.632 mg; vitamin D3—0.1232 mg; vitamin E—23.584 mg; vitamin K3—3.52 mg; copper—16.00 mg; iron—100.00 mg; iodine—2.40 mg; manganese—140.00 mg; selenium—0.60 mg; zinc—140.00 mg.

**Table 2:** Composition of experimental diets by supplementation technique (g/kg).

Items (g/kg)	D1	D2	D3	D4	D5	D6	D7	D8
Basal diet	1000	991.439	991.462	991.484	991.505	991.527	991.548	991.582
L-glutamic acid 98%		2.245	1.971	1.698	1.425	1.152	0.879	0.460
Starch		0.980	0.960	0.939	0.919	0.899	0.879	0.848
Kaolin		5.086	5.107	5.129	5.151	5.172	5.194	5.227
L-tryptophan biomass 60%		0.250	0.500	0.750	1.000	1.250	1.500	1.883
Total	1000	1000	1000	1000	1000	1000	1000	1000

D: diet.

**Table 3:** Treatments, levels, and ideal ratio tryptophan/lysine.

Items (%)	D1	D2	D3	D4	D5	D6	D7	D8
Ideal ratio tryptophan/lysine	15	17	19	21	23	25	27	30
L-tryptophan in the diet	0.112	0.127	0.142	0.157	0.172	0.187	0.202	0.225

D: diet.

**Table 4:** Average responses of daily feed intake (FI, g/hen), daily egg production (EP, %), egg weight (EW, g), daily egg mass (EM, g/hen), feed conversion ratio (FCR, g/g), and feed efficiency (FE, g/kg) of hens fed with tryptophan in the diet (TRP, %) from 70 to 86 weeks of age.

Treatments	TRP	FI	EP	EW	EM	FCR	FE
D1	0.112	108.2	81.4	59.2	48.2	2.251	445.1
D2	0.127	107.7	84.6	60.9	51.5	2.093	478.7
D3	0.142	108.4	88.5	60.3	53.3	2.036	491.7
D4	0.157	108.9	92.8	60.1	55.8	1.954	512.3
D5	0.172	108.7	94.1	59.8	56.3	1.932	518.5
D6	0.187	109.0	94.6	60.0	56.7	1.924	520.3
D7	0.202	107.7	93.8	59.4	55.7	1.937	516.8
D8	0.225	108.0	93.1	59.3	55.2	1.957	511.3
Average		108.4	90.6	59.8	54.2	2.008	500.0
SEM		0.2	0.5	0.2	0.3	0.012	3.0
CV, %		1.9	5.8	3.3	6.1	6.1	5.8
*p*-value							
Degree of freedom		98	98	98	98	98	98
ANOVA		0.329	<0.0001	0.507	<0.0001	<0.0001	<0.0001
Linear effect		0.949	<0.0001	0.307	<0.0001	<0.0001	<0.0001
Quadratic effect		0.108	<0.0001	0.224	<0.0001	<0.0001	<0.0001

D: diet. SEM: Std error of mean. CV: coefficient of variation.

**Table 5:** Average of yolk weight (YW, g), eggshell weight (ESW, μm), albumen weight (AW, g), eggshell thickness (EST, μm), percentage yolk (PY, %), percentage albumen (PA, %), and percentage shell (PS, %) of hens fed with tryptophan in the diet (TRP, %) from 70 to 86 weeks of age.

Treatments	TRP	YW	ESW	AW	EST	PY	PA	PS
D1	0.112	15.4	5.9	37.8	400.9	26.0	64.0	9.9
D2	0.127	15.7	6.2	39.0	404.8	25.7	64.1	10.2
D3	0.142	15.4	6.1	38.9	407.6	25.5	64.4	10.1
D4	0.157	15.5	6.1	38.5	407.4	25.8	64.0	10.1
D5	0.172	15.5	5.9	38.4	398.9	25.9	64.2	9.9
D6	0.187	15.2	6.0	38.7	400.7	25.4	64.6	10.0
D7	0.202	15.3	6.0	38.2	401.1	25.8	64.3	10.0
D8	0.225	15.4	5.9	38.1	401.2	26.0	64.1	10.0
Average		15.4	6.0	38.4	403.1	25.7	64.2	10.0
SEM		0.1	0.03	0.2	1.2	0.1	0.1	0.04
CV, %		3.8	4.5	4.3	3.0	3.4	1.6	3.8
*p*-value								
Degree of freedom		98	98	98	98	98	98	98
ANOVA		0.595	0.138	0.772	0.635	0.693	0.925	0.798
Linear effect		0.342	0.247	0.605	0.349	0.956	0.547	0.640
Quadratic effect		0.955	0.124	0.213	0.563	0.220	0.463	0.455

D: diet. SEM: Std Error of mean. CV: Coefficient of variation.

**Table 6:** Average responses of body weight (BW, g/hen), change in body weight (cBW, g/hen), body water (Water, g/hen), body protein (Protein, g/hen), body lipid (Lipid, g/hen), and body ash (Ash, g/hen) of hens fed with tryptophan in the diet (TRP, %) from 70 to 86 weeks of age.

Treatments	TRP	BW	cBW	Water	Protein	Lipid	Ash
D1	0.112	1810	155.0	913.5	274.3	177.0	56.6
D2	0.127	1808	155.0	870.4	261.1	165.3	55.1
D3	0.142	1838	149.0	879.7	261.3	179.1	54.5
D4	0.157	1817	143.0	980.8	291.4	184.5	60.6
D5	0.172	1852	150.0	906.9	269.4	184.6	56.1
D6	0.187	1856	144.0	896.4	268.9	166.2	56.7
D7	0.202	1820	87.0	915.7	272.0	166.7	56.7
D8	0.225	1795	129.0	902.3	268.0	166.3	55.8
Average		1830	136.0	912.9	272.1	173.7	56.8
SEM		9.0	8.0	5.5	1.6	1.1	0.3
CV, %		5.0	5.9	6.0	5.9	6.0	5.8
*p*-value							
Degree of freedom		98	98	98	98	93	98
ANOVA		0.474	0.476	<0.0001	<0.0001	<0.0001	<0.0001
Linear effect		0.965	0.100	0.528	0.879	0.0007	0.729
Quadratic effect		0.104	0.911	0.130	0.211	<0.0001	0.097

D: diet. SEM: Std Error of mean. CV: Coefficient of variation.

**Table 7:** Estimated parameters of the linear–plateau (LP) and quadratic–plateau (QP) models of the variables of daily egg production, daily egg mass, feed conversion ratio, and feed efficiency relationship between daily tryptophan intake of hens from 70 to 86 weeks of age.

Variables (γ)	Linear–Plateau (LP)					Quadratic–Plateau (QP)					^1^QP_1_–LP
	φ	β	λ	R^2^	AIC	ξ	β	τ	R^2^	AIC	
Daily egg production, %	94.4	−0.22	180.9	78.0	343.3	94.4	−0.002	207.3	78.0	347.6	186.2
Daily egg mass, g/day	56.1	−0.18	168.4	78.3	267.0	56.2	−0.002	188.9	79.0	269.1	174.7
Feed conversion ratio, g/g	1.921	0.006	182.1	81.1	−194.3	1.923	0.00005	203.0	80.9	−192.7	182.7
Feed efficiency, g/kg	520.2	−1.26	182.5	80.2	562.6	520.2	−0.011	206.3	80.0	566.6	182.5

γ: Response variable; φ: is maximum response; β: is the slope; λ: is requirement for maximum response (mg/hen); R^2^: coefficient of determination; AIC: Akaike information criterion; ξ: is maximum response; τ: is requirement for maximum response (mg/hen); ^1^QP_1_–LP, 1st interception of QP on the plateau of the LP.

**Table 8:** Partitioning of tryptophan intake to maintenance and egg deposition according to tryptophan in the diet (TRP, %) of hens fed from 70 to 86 weeks of age.

Treatments	TRP	Tryptophan Intake	Tryptophan Deposited in Egg	Tryptophan Maintenance
D1	0.112	121.2	91.0	15.5
D2	0.127	136.7	97.4	15.5
D3	0.142	153.9	100.7	15.6
D4	0.157	171.0	105.4	15.5
D5	0.172	186.9	106.5	15.7
D6	0.187	203.9	107.2	15.7
D7	0.202	217.6	105.2	15.5
D8	0.225	243.0	104.3	15.4
Average		180.2	102.398	15.6
SEM		3.7	0.63	0.1
CV, %		20.6	6.1	3.4
*p*-valor				
Degree of freedom		98	98	98
ANOVA		<0.0001	<0.0001	0.473
Linear effect		<0.0001	<0.0001	0.944
Quadratic effect		0.106	<0.0001	0.103

D: diet. SEM: Std Error of mean. CV: Coefficient of variation.

## Data Availability

The data presented in this study are available on request from the corresponding author.

## Abbreviations

The following abbreviations are used in this manuscript:

5-HT	Serotonin
AIC	Akaike information criterion
AW	Albumen weight
BD	Basal diet
BL	Broken-line
BW	Body weight
cBW	Change body weight
CV	Coefficient of variation
D	Tryptophan deposition in egg
DAHP synthase	3-deoxy-D-arabino-heptulosonate-7-phosphate synthase
DEXA	Dual-energy X-ray absorptiometry
E4P	Erythrose-4-phosphate
EM	Egg mass
EP	Egg production
EST	Eggshell thickness
ESW	Eggshell weight
EW	Egg weight
FCR	Corrected for mortality
FI	Feed intake
I	Trp intake
IPA	Indole-3-pyruvic acid
k	Trp utilization efficiency
k_e_	Trp utilization in egg deposition
k_t_	Efficiency of Trp utilization
LL	Linear–linear
LP	Linear–plateau
m	Trp maintenance
PA	Percentage albumen
PEP	Phosphoenol–pyruvate
PS	Percentage shell
PY	Percentage yolk
QP	Quadratic–plateau
R^2^	Coefficient of determination
Trp	Tryptophan
YW	Yolk weight
